# Barley landraces are characterized by geographically heterogeneous genomic origins

**DOI:** 10.1186/s13059-015-0712-3

**Published:** 2015-08-21

**Authors:** Ana M. Poets, Zhou Fang, Michael T. Clegg, Peter L. Morrell

**Affiliations:** Department of Agronomy and Plant Genetics, University of Minnesota, St. Paul, MN 55108 USA; Current address, Bayer CropScience, 407 Davis Drive, Morrisville, NC 27560 USA; Department of Ecology and Evolutionary Biology, University of California, Irvine, CA 92697 USA

## Abstract

**Background:**

The genetic provenance of domesticated plants and the routes along which they were disseminated in prehistory have been a long-standing source of debate. Much of this debate has focused on identifying centers of origins for individual crops. However, many important crops show clear genetic signatures of multiple domestications, inconsistent with geographically circumscribed centers of origin. To better understand the genetic contributions of wild populations to domesticated barley, we compare single nucleotide polymorphism frequencies from 803 barley landraces to 277 accessions from wild populations.

**Results:**

We find that the genetic contribution of individual wild populations differs across the genome. Despite extensive human movement and admixture of barley landraces since domestication, individual landrace genomes indicate a pattern of shared ancestry with geographically proximate wild barley populations. This results in landraces with a mosaic of ancestry from multiple source populations rather than discrete centers of origin. We rule out recent introgression, suggesting that these contributions are ancient. The over-representation in landraces of genomic segments from local wild populations suggests that wild populations contributed locally adaptive variation to primitive varieties.

**Conclusions:**

This study increases our understanding of the evolutionary process associated with the transition from wild to domesticated barley. Our findings indicate that cultivated barley is comprised of multiple source populations with unequal contributions traceable across the genome. We detect putative adaptive variants and identify the wild progenitor conferring those variants.

**Electronic supplementary material:**

The online version of this article (doi:10.1186/s13059-015-0712-3) contains supplementary material, which is available to authorized users.

## Background

The domestication of plants and animals around 10,500 YBP initiated the development of complex human societies and provided the raw material on which modern agriculture still depends [1–3]. Barley and early forms of wheat, and later pea, lentil, chickpea, and a number of other species were the primary plants in the Neolithic agropastoral package that originated in the Fertile Crescent and later spread across North Africa and most of Eurasia [4, 5]. A growing body of archeological evidence suggests that Fertile Crescent agriculture involved a gradual transition from plant collection into management and cultivation [2, 6–8]. Having started with the collection of seed from fully wild barley populations that began as much as 50,000 YBP [5, 9] agricultural practices were ultimately widely disseminated through a mix of cultural and demic diffusion [4, 6, 10, 11].

Extensive archeological remains at human Neolithic sites capture the timing and phenotypic transition from wild to cultivated barley across the Near East [2, 5, 8, 12, 13] making barley a particularly desirable system to study the evolution of domestication. The biology of the species also facilitates genetic studies because it is a diploid, self-fertilizing species with a genetically diverse wild progenitor that has a broad geographic distribution [2] marked by substantial genetic differentiation among wild populations [14]. Recent genetic studies of wild and landrace (primitive domesticate) barley collections [15, 16] and evidence of independent origins of important domestication-related traits [17–19] support the hypothesis of at least two independent domestication events followed by some degree of admixture among domesticates from distinct portions of the geographic range of the wild barley distribution. This scenario is also consistent with minimal loss of diversity in cultivated barley relative to its wild ancestor [20]. Here we address the following questions: (1) Do specific wild populations contribute disproportionately to barley landraces? and (2) does the genetic contribution of wild populations to landraces vary across the genome or across the broad geographical range of landrace cultivation?

Multiple lines of evidence, presented here, indicate that barley landraces have mosaic ancestry, reflecting the contribution of all major geographic portions of the range of the wild progenitor species. A broad contribution of wild progenitor populations to the landraces is consistent with archeological evidence for a gradual transition to cultivation [21]. This is demonstrated by phenotypic change, particularly non-shattering of the inflorescence, which is essential for barley domestication, identified at many Neolithic sites. Identification of putatively adaptive contributions from wild progenitor populations provides a potential means of detection of loci contributing to locally adaptive variation (for example, for climatic adaptation).

## Results and Discussion

### Population structure and genetic differentiation among barley landraces

**Fig. 1:**
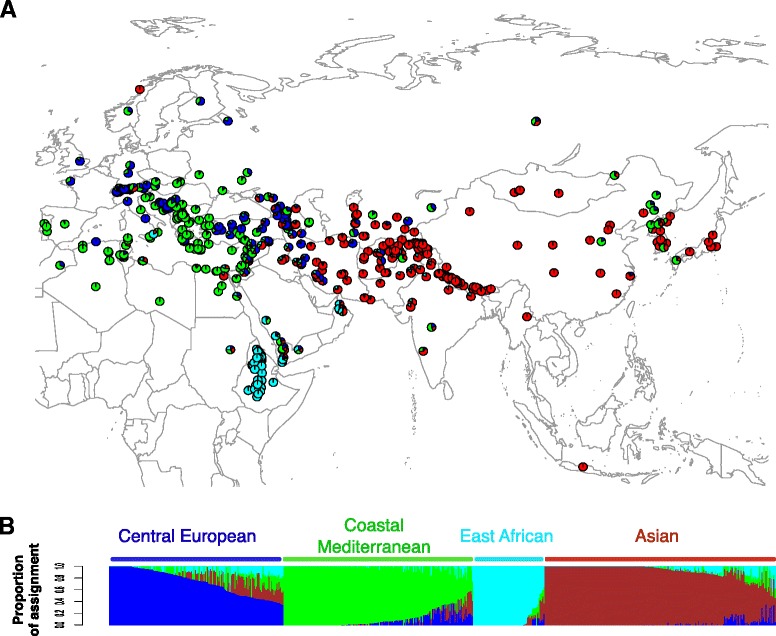
Distribution and populations structure of the barley landraces in the Old World used in this study. **a** Colors correspond to the proportion of assignment to each of the four populations identified among landraces. **b** Secondary population subdivision for the optimal *K* = 4: Central European, Mediterranean, East African, and Asian. Each color represents the majority of assignment for four landrace populations. The Y-axis is percent composition with samples sorted along the X-axis geographically by longitude from west to east

To investigate the contribution of wild to domesticated barley we first examined the extent of population structure among landraces using genotyping data from 6,152 SNPs in 803 landrace accessions collected in Europe, Asia, and North Africa (Fig. 1a, Additional file 1). Population structure was estimated using a Bayesian clustering algorithm implemented in STRUCTURE [22]. Four major groups of landraces were identified: Coastal Mediterranean, Central European, East African, and Asian (Fig. 1b, see Table 1 for summary statistics of these populations). The first three groups are nested within a Western primary population (when *K* = 2) while Asian landraces correspond to the Eastern partition (Additional file 2), similar to the structure reported in previous studies [15, 16, 20]. The genetic assignment results agree with estimates of the degree of differentiation among landrace individuals by Principal Component Analysis (Additional file 3), and with the genetic differentiation identified by *F*-statistics [23] (see Additional file 4 for a summary of pairwise *F*_ST_ comparisons). In summary, western wild barley populations appear to contribute most directly to the genetic constitution of African and European landraces, while eastern wild barley populations made a greater contribution to Asian landraces.

**Table 1:** Summary statistics for the four landrace populations, based on 6,152 SNPs

Landraces	Sample size	Segregating sites	Private alleles	Pairwise diversity
Central European	210	6,004	70	0.337
Asian	279	5,541	26	0.268
Coastal Mediterranean	228	5,950	40	0.309
East African	86	4,298	3	0.210

Values for sample size, number of segregating sites, number of private alleles, and percent pairwise diversity scaled by number of segregating sites are reported

### Inference of the genetic contribution of wild populations at specific genomic segments

**Fig. 2:**
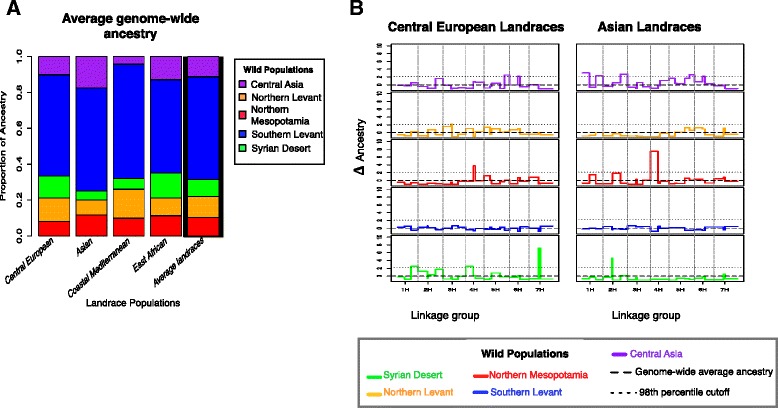
Proportions of genetic ancestry. **a** Genome-wide proportion of ancestry for each landrace population. Colors represent the population of origin from the wild (Unassigned sites are not considered). The barplot on the far right (inside a black box) represents the average genome-wide ancestry among all four landrace populations, used also as a base line for panel **b** (average contribution). **b** Excess or deficit of ancestry (Δ ancestry) for the Central European and Asian landrace populations. Δ ancestry is measured as the deviation from the average contribution of each wild population at the genome-wide level (that is, how many times more/less of that ancestry is observed at each genomic segment with respect to genome-wide ancestry proportion) (black dashed line). Colors correspond to the five populations identified in wild barley (Additional file 5). Positive values indicate X times of excess ancestry and negative values a deficit of ancestry with respect to the genome-wide average ancestry of a particular wild population. The dotted horizontal line indicates the 98th percentile cutoff from the distribution of excess or deficit of each wild population across all genomic segments at each landrace population

Beyond evidence for the primary genetic composition and origins of landraces, there are more subtle patterns of genetic exchange. Each of the populations identified in wild barley [14] (Additional file 5) contributes to the genetic composition of the four landrace populations, but this contribution is heterogeneous across genomic segments (Fig. 2b, Additional files 6 and 7). This is demonstrated by an analysis of admixture, based on genetic assignment using five of the six wild barley populations as learning samples. These are used to identify the contribution of each wild barley population to individual genomic segments in the landrace populations (see Materials and Methods for the rationale for removing the Caspian Sea wild population from the learning sample). This analysis is based on SupportMix, a tool designed to examine admixture proportions across the genome [24]. The analysis is focused on 75 SNP windows because this window size maximized assignment probabilities while permitting the comparison of a large number of genomic segments. Only 17.6 % of genomic segments have a probability of assignment below 0.95, and are marked as missing data (unassigned, Additional file 8). The genome-wide proportion of ancestry is estimated as the proportional contribution of each wild population to all landraces (Fig. 2a, Additional file 9). We then estimated the excess or deficit of ancestry (referred to here as Δ ancestry) for each genomic segment in each landrace population. Δ ancestry is the difference between the contributions from each wild population for a particular genomic segment to the average genome-wide proportion of ancestry derived from that wild population (Fig. 2b, Additional file 6). The predictive accuracy of this approach was evaluated by using accessions from the wild barley populations to assign individual genomic segments relative to their known population of origin (cross-validation). This analysis indicates that the power of SupportMix to infer ancestries at any given genomic segment (that is, the potential to accurately assign an individual back to a known population of origin) in our dataset averages 69 % (among genomic segments with probability of assignment 0.95) (Additional files 10 and 11). This value, although slightly lower than previously reported values of robustness for estimators of ancestry of genomic segments (approximately 80 %) [25], is consistent with the challenge of resolving the contribution of five possible source populations for each genomic segment across all 803 landrace accessions in our sample.

Across all landrace populations, for the fraction that had 0.95 probability of assignment (82.5 %), the largest genome-wide proportion of ancestry derives from the Southern Levant wild population (57 %) (Additional files 9 and 12). These results agree with previous archeological and genetic data that identified the Southern Levant (present-day Israel) as the primary contributor to domesticated barley [9]. Higher assignment to wild barley from the western portion of the range (particularly the Southern Levant) is also expected due to greater representation of SNPs discovered in this region on the genotyping platform [14, 26]. Along with the Southern Levant contribution, the genetic composition of landrace populations reflects an average contribution of 12 % from Northern Levant, 11 % Central Asian, 10 % Northern Mesopotamia, and 9 % Syrian Desert wild populations (Fig. 2a). Although, the average genome-wide ancestry among landrace populations is similar (Fig. 2a), the within population variation indicates that the contribution from wild populations differs among individuals in a population (Additional file 12). Moreover, the genetic composition of landrace populations varies across genomic regions (Fig. 2b, Additional file 6). The indication that multiple wild populations contributed to current genetic composition is similar to the patterns observed for domesticated emmer wheat [27]. The East African landrace population is inferred to have highly admixed ancestry from multiple wild barley populations (Additional file 6D). This is consistent with earlier conjecture that barley was imported to Ethiopia from domesticated sources [28].

### There is a higher genetic contribution from proximate wild populations into landraces

**Fig. 3:**
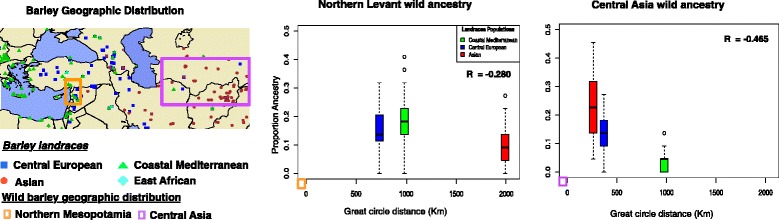
Genome-wide ancestry as a function of distance from wild populations. The map on the left indicates the distribution of landraces sympatric with populations of wild barley. The orange and purple boxes represent the geographic distribution of Northern Levant and Central Asian wild populations. The panels on the right indicate the distribution of proportion of ancestry (Y-axis) in each of the landrace populations as a function of distance (X-axis) from the source wild population with the closest proximity. The boxplot for each landrace population was placed at the population’s median value of the great circle distance between each landrace individual to the closest wild barley individual in the wild barley population analyzed (depicted at coordinates 0,0). The correlation (*r*) between distance and proportion of ancestry is indicated in each comparison. East African landraces are not included in the depiction due to small sample size (two individuals) in the geographic range analyzed

There is abundant archeological evidence of human mediated movement and dissemination of cultivated barley beyond the initial range of domestication, beginning approximately 8,000 YBP [4, 5]. In addition, our study shows that landraces frequently carry genomic segments with inferred ancestry that most closely resembles proximate wild populations (Fig. 2b and Additional file 6). For example, a higher contribution of proximate wild populations is evident at 13 % (4/29) of the genomic segments in Asian landraces (Fig. 2b), with an excess of ancestry derived from the Central Asian wild population compared to the average landrace ancestry genome-wide. The proportional contribution of wild populations to proximate landraces is reflected in greater genome-wide similarity relative to great circle distance from the neighboring wild population (Fig. 3, Additional files 13 and 14). This is evident in a negative correlation (*r*) between geographic distance and genetic contribution of the Central Asian wild population to the Asian landraces (Fig. 3, Additional file 14) (*r* = -0.47). A similar pattern is observed in all other comparisons between the proportion of ancestry and distance from each wild population. This correlation is consistent with isolation by distance, with *r* equal to -0.28 and -0.27 for comparisons to Northern Levant and Syrian Desert wild populations, respectively. There is very limited correlation with distance (0.04) for Northern Mesopotamia and Southern Levant populations. Within each landrace population, individual samples have distinct genetic compositions, with some accessions carrying higher proximate wild ancestries than the average in their population. For example, the Northern Mesopotamia wild population contributed 12 % of the genomic segments in Asian landraces (Additional files 8 and 9), but variance among individuals results in > 20 % contribution to some individual landrace accessions in this population (Additional file 9).

### Private alleles provide direct evidence of contributions of wild populations to landraces

The frequency of SNPs in the landraces that are unique (private) to any of the wild populations (181 SNPs total, Additional file 15) is examined to further delineate the contribution of individual wild populations to the genetic composition of barley. We find 127/161 (79 %) of alleles private to Western (as opposed to Eastern) wild populations present in Asian landraces at an average frequency of 19.5 %. There are 18/20 (90 %) of alleles private to the Eastern wild population present in the Coastal Mediterranean and Central European landrace populations with an average frequency of 24 % (Additional files 16 and 17). The larger number and frequency of private alleles from Western wild populations present in Asian landraces are consistent with the genetic assignment analysis reported above. This indicates a greater contribution of Western wild barley to Asian landraces than Eastern wild barley to Coastal Mediterranean and Central European landraces, consistent with previous results based on resequencing [15, 20]. The private/shared allele comparison also identifies a greater contribution of Southern Levant private alleles to all landrace populations (Additional files 15 and 16). The higher contribution from the Southern Levant wild population should be treated as preliminary, as ascertainment bias could influence our observations. Using coalescent simulations, Fang *et al*. [29] found that the discovery panel for this set of SNPs is best modeled as derived from eight inbred lines, retaining variants with a minimum of three occurrences in the discovery panel. This accords well with the discovery scheme reported by Close *et al*. [30], which includes an eastern wild barley and Japanese cultivar, but is generally weighted toward European and North American barley cultivars where the genetic composition is contributed largely by western wild populations [15, 20]. When using private alleles to estimate the contribution from other wild populations, this will have the (conservative) effect of underestimating the contribution of other wild populations to landraces.

There is an uneven genetic representation of wild populations across various landrace populations at specific genomic regions (Additional file 17), perhaps suggesting that particular adaptations have been combined in landraces from geographically diverse wild populations. This is evident, for example, in the higher frequency (76.6 %) of SNP variant 11_21184 (linkage group 2H) private to Northern Mesopotamia wild populations found in all landrace populations except in the Coastal Mediterranean (Additional files 15 and 17). A similar pattern is observed for SNP variant 11_10480 (linkage group 4H) that is private to the Syrian Desert wild population, but is found in high frequency (81.3 %) in all landrace populations except for the Asian population (Additional files 15 and 17).

The increased genetic resemblance between landraces and proximate wild populations indicates the potential adaptive nature of alleles found in genomic segments with higher positive Δ ancestry, or high frequency private alleles. For example, Δ ancestry values indicate regions on linkage groups 1H, 2H, and 5H in the Asian landraces (Fig. 2b, Additional file 6B) that have an elevated contribution from the Central Asian wild population. Although, this excess cannot be explained solely by the presence of Central Asian private alleles, there is one Central Asian wild private SNP variant 11_21286 (linkage group 2H) at 63 % frequency in Asian landraces and virtually absent or in low frequency in the Coastal Mediterranean, Central European, and East African populations (Additional files 15 and 17). Likewise, Coastal Mediterranean, Central European, and East African populations have a higher proportion of Northern Mesopotamia ancestry (Δ ancestry) at two genomic segments at linkage group 4H (Additional file 6). We identify two SNP variants private to Northern Mesopotamia (11_10756 and 12_30136) at high frequency (77 %) in the middle segment on linkage group 4H (Additional file 15). The private/shared alleles analysis also confirms the admixed nature of the East African population, yet with a larger contribution from Western wild populations. The East African population includes 12 private alleles derived from Eastern wild populations (33 % frequency) and 103 private alleles from Western wild populations (44 % frequency) (Additional file 16).

### Similarity between wild and landrace populations cannot be explained by recent introgression

An alternative hypothesis for the mosaic ancestry of landraces involves recent or ongoing introgression from proximate wild populations [15]. Population genetic effects of recent introgression include large chromosomal regions in (admixture) linkage disequilibrium (LD) [31–33] or extended genomic tracts of shared ancestry [34, 35]. Admixture LD breaks down quickly in outcrossing species, but should be more readily detectable in self-fertilizing species such as barley. The estimated rate of outcrossing 1.8 %, averaged across samples of wild and cultivated barley [36] should greatly reduce the rate of effective recombination [see reference 37], dramatically increasing the number of generations for the decay of admixture LD. An analysis of identity by state (IBS) among the landraces and wild barley populations conditioning on a complete match over 30 SNPs (which constitutes approximately 1/15 of the SNPs per linkage group) identifies 37 non-overlapping IBS segments (Additional file 18). Only 18 % of wild and 36 % of landrace individuals contribute to this perfect-match IBS (Additional file 19) whereas differential ancestry for individual genomic segments can involve >80 % of landraces (for example, from Northern Mesopotamia wild population in the first genomic segment in linkage group 4H in Asian landraces; Additional file 8B). Some degree of IBS is expected among distantly related individuals from distinct populations, owing to the expectations of deep patterns of shared descent within a species [38]. IBS comparisons fail to identify large shared segments (constituting half or one-quarter of linkage groups), as expected after introgression [33]. The low levels of genome-wide LD (Additional file 20) and small blocks of IBS (average 10.5 cM) suggest that contributions from wild populations into the cultigen are not recent and in some cases may date back to early in the history of widespread barley cultivation which started around 8,500 YBP [1, 2, 5, 12].

## Conclusions

In summary, the genetic composition of barley landraces indicates a genetic contribution from multiple wild progenitor populations that in turn must reflect the pattern of initial domestication and later patterns of trade and migration of early agriculturalists along the axes of Europe, Africa, and Asia. Although multiple populations contribute to the genetic composition of the cultigen, the contribution from the broad geographic range of wild barley populations also varies across the genome as well as across landrace populations. The clear contribution from proximate wild populations, at specific genomic regions, raises the intriguing possibility of adaptive contributions based on regional and local environments.

## Materials and Methods

We used 803 barley landrace accessions from the 2,446 landrace and cultivated lines in the National Small Grains Collection (NSGC) Core Collection from the USDA. These 803 individuals include all landraces collected in Europe, Asia, and North Africa constituting the range of dissemination of cultivated barley in human pre-history (Fig. 1, Additional file 1).

We also make use of the 284 wild barley accessions from the Wild Barley Diversity Collection (WBDC) [39] analyzed in [14]. Accessions represent the entire geographic range of wild barley including the Fertile Crescent, Central Asian, and adjacent North African regions.

### Genotypic data

A collection of 2,446 landraces and cultivated accessions from the NSGC were genotyped with 7,864 SNPs using the Illumina Infinium SNP genotyping platform (hereafter referred to as the 9K). The 9K chip contains 5,010 SNPs discovered in a panel of 10 barley varieties, composed primarily of European two row cultivars. In addition, a set of 2,832 SNPs used for the existing BOPA (Barley Oligo Pooled Assay 1 and 2) on the Illumina Golden Gate genotyping platform [30] was included. Additionally, 22 SNPs from resequencing studies were added, giving a total of 7,864 SNP assays on the chip [40]. The BOPA SNPs derived principally from one wild barley accession and eight malting barley cultivars, from Europe, the United States, and Japan [30].

We used automated genotype calling implemented in the software ALCHEMY [41]. ALCHEMY uses a Bayesian model of the raw intensity data files. This approach does not assume Hardy-Weinberg Equilibrium; and each single nucleotide polymorphism (SNP) call is independent of other genotype calls at the SNP. SNP calls with posterior probability >0.95 were recorded; calls below this threshold were marked as missing data. The accuracy of calls was verified following the method explained previously [14].

SNP quality control procedures consisted of the removal of SNPs that were monomorphic, had more than 10 % missingness, or had more than 10 % heterozygosity [see reference 14]. We retained 6,152 SNP for all 2,446 landraces and cultivated lines after quality control. The curated SNP dataset was used to identify potential duplicate individuals in the NSGC barley core. The details of the procedure used to identify duplicate accessions are explained in Muñoz-Amatriaín *et al*. [42]. We retain 803 landrace accessions after quality control.

The 284 accessions from the WBDC were genotyped with 3,072 SNPs [30], a subset of the 9K platform. After quality control this dataset consisted of 2,624 SNPs for each of the 284 accessions [see reference 14] for specific information about these populations and SNP quality control steps.

We used the consensus genetic map described in [42] which is the result of merging the 11 genetic maps of the 2011 consensus map developed by Muñoz-Amatriaín *et al*. [43] with the iSelect SNP platform map based on the Morex x Barke mapping population [40]. This map, referred to here as the ‘iSelect map’, identifies genetic position for 4,527 of the SNPs used to genotype the NSGC accessions.

We infer the phase of heterozygous sites (approximately 0.1 % of sites) using PHASE v.2.1.1 [44, 45] for all 1,896 SNPs which were shared between landraces and wild barley, and had genetic map positions (Additional file 21). The runs are set to the default values for number of iterations = 100, thinning intervals = 1, and burn-in = 100. We consider only phased calls with probabilities of at least 90 %. All imputed sites for missing data are re-set to missing values using a customized R script (R Project for Statistical Computing, http://www.r-project.org/). Experimentally phased haplotypes are used in two analyses where they are critical to inference, that is, the estimation of admixture proportions and assessment of identity by state between wild and landrace accessions.

Linkage disequilibrium (LD) as measured by *r*^2^ [46] is calculated for all possible pairwise comparisons on each linkage group based on the 4,527 SNPs included in the iSelect genetic map. We considered SNPs with minor allele frequency (MAF) >5 %. The LDheatmap package in R [47] was used to generate plots of LD relative to genetic distance (Additional file 20).

### Genetic assignment

To determine the geographic population structure among the 803 landraces in our dataset, we used a Bayesian clustering algorithm implemented in STRUCTURE [22, 48]. We explored the numbers of clusters (referred to as *K*) ranging from 1 to 7 (Additional file 2). For each value of *K* we used 10 replicated runs, with a burn-in length and run length of 100,000 iterations. We used an admixture model because archeological and genetic evidence suggest extensive movement of barley and thus likely admixture [4, 5, 15, 20, 49]. We used the uncorrelated allele frequency model, which is more conservative. STRUCTURE analysis was run based on the 6,152 SNPs for the 803 landraces. Considering the high selfing rate of barley >98.2 % [36] we used a haploid model (option PLOIDY=1). To summarize the assignment results for all replications we used CLUMPP [50]. CLUMPP deals with label switching (that is, when cluster names change between replicates); and multimodality (that is, when individual samples change clusters in each replication).

We used two *ad hoc* approaches, Δ*K* [51] and Clusterdness [52], to determine the number of clusters that best explain the population structure among the landraces. Δ*K* is based on the second order rate of change of the log probability of data between successive *K* values [51], and Clusterdness [52] is the extent to which individuals are estimated to belong to a single cluster rather than to a combination of clusters (Additional file 22).

The primary population structure identified here (*K* = 2, Additional file 2) agrees with previous observations of population differentiation of landrace and wild barley accessions east and west of the Zagros Mountains [15, 20]. The large sample considered here permits greater resolution of the geographic differentiation among barley landraces (Additional file 2).

We estimated the degree of differentiation among individuals by PCA. For this analysis we use all the 4,527 SNPs with known genetic position for the 803 landrace accessions. The PCA was performed in the SmartPCA program from the EIGENSOFT package [53]. SmartPCA permits PCA analysis with SNP loci that include missing data, thus our analysis is based on the full SNP genotyping dataset. Procrustes analysis [54] implemented in the vegan package in R [55] was used to identify the optimal rotation that maximizes the similarity between genetic variation on PCA plot and geographic maps of sample locations (Additional file 3).

We used SharedPoly and compute from the libsequence library [56] to calculate summary statistics, including number of segregating sites, number of private alleles in each cluster, and the percent pairwise diversity scaled by number of segregating sites (Table 1).

To further analyze the degree of differentiation between these populations we calculated *F*-statistics [23, 57] for individual SNPs (6,152 SNPs) genome-wide implemented in the R package HierFstat [58]. To detect genetic differentiation in individual groups of landraces we used focal comparisons of each population to the overall dataset (Additional file 4).

### Admixture inference

Using a Maximum Likelihood approach implemented in TreeMix [59], we infer the patterns of population split and mixtures between the six wild barley populations identified in Fang *et al*. [14]. Populations were identified as Caspian Sea (seven accessions), Central Asian (53 accessions), Northern Levant (42 accessions), Northern Mesopotamia (41 accessions), Southern Levant (107 accessions), and Syrian Desert (34 accessions) (see Table S1 in Fang *et al*. [14], for geographic location of WBDC accessions). The TreeMix analysis included all wild populations and the four landrace populations identified here. We ran 25 replications of the tree without bootstrapping, and 25 replications with bootstrapping including five SNPs and 25 SNPs at a time. From this we determine that the wild population from the Caspian Sea is more closely related to landrace populations than to other wild populations (Additional file 23) thus suggesting the possibility of a more recent introgression with the landraces [60]. Including the Caspian Sea wild population in the ancestry analysis of the landraces results in greater contribution from the Caspian Sea wild population than expected based on historical human migration information (data not shown). Although, the Caspian Sea wild individuals resemble wild barley morphologically (with a shattering inflorescence and extensive branching) other traits such as seed size and erect tillers could suggest either convergent evolution of phenotypes or that the Caspian Sea wild population has been in more recent contact with domesticated material, a result which could potentially bias our inferences of ancestry. Based on this observation the seven individuals from the Caspian Sea wild population are excluded from analysis of population ancestry, retaining 277 wild barley accessions. We note here that the original WBDC encompasses 318 accessions. Fang *et al*. [14] identified 30 accessions that appear to be duplicated within the sample or have genotypic composition suggestive of recent introgression. These along with four other samples were removed from the study due to missing latitude and longitude information, resulting in 284 wild barley accessions in our sample.

We utilized a machine learning approach implemented in the software SupportMix [24] to identify the contribution of each of the wild barley populations to individual genomic segments in the landraces. SupportMix can perform admixture analysis by simultaneously analyzing a large number of possible source populations, regardless of their relationship to the focal population and without making assumptions regarding population demographic history or specific population genetic parameters [24]. SupportMix is a two-level method. First, it uses a support vector machine for the classification of the ancestral populations at each genomic segment independent of each other. Once the model is trained to distinguish the source populations it takes one sample at a time (for a specific genomic segment) and assigns it to a putative source population. Second, after all genomic segments are classified for each accession it continues with a smoothing step using a Hidden Markov Model to detect transitions between the different ancestral groups, this approach considers correlations between genetic blocks to limit the effect of regions with poor information content. The wild population with the highest genetic similarity is assigned as the source for that genomic segment and given a probability of assignment to that source population.

The five wild barley populations identified as clearly distinct groups from the landraces are used as potential source populations for the landraces. For this analysis we used 1,896 SNPs found on the iSelect genetic map that were common between the wild barley (SNPs are polymorphic in all 277 wild accessions) and the collection of landraces (803 individuals) (Additional file 21). We ran SupportMix on genomic segments comprised of 50, 75, and 100 SNPs. The wild population with highest similarity is assigned as the source population for that segment. Individual assignment probabilities below 95 % are treated as missing. Inference of admixture using 50 SNP windows results in a large proportion (45 %) of genomic segments with probabilities of assignment below our threshold of 0.95. Thus, SNP windows shorter than 50 SNPs are not used. Increasing the window size to 75 and 100 SNPs results in a higher confidence of ancestry assigned for each genomic region. In these two analyses there were 17.5 % and 19.5 % of the genomic segments across the seven linkage groups in our sample of landraces with probability of assignment below 0.95, respectively. These segments coincide primarily with the boundaries of linkage groups and are treated as missing data. Therefore we use windows of 75 SNPs. The proportion of ancestry genome-wide is estimated as the percentage of contribution of each wild population to the complete landraces dataset.

The predictive accuracy of SupportMix for genetic assignment of individual genomic segments was evaluated by cross-validation using a subset of wild barley individuals as testing samples, maintaining the remaining wild accessions as the validation sample. The test was run 50 times, sampling four accessions (eight haplotypes) from each wild population per iteration, without replacement. As in the landrace assignment, we used windows of 75 SNPs. An average of 16.4 % of genomic segments per individual could not be assigned with confidence (probability of assignment <0.95) to any population of origin. Window sizes smaller than 75 SNPs resulted in > 80 % of the genome being unassigned (data not shown). In summary, among genomic segments that are assigned with high confidence, 69 % are correctly assigned to the population of origin. A notable exception to assignment of genomic segments of wild individuals back to population of origin occurred in the Northern Levant population, where proportional assignment to the Northern Levant wild population averaged 43 %, with 31 % of segments assigning to the geographically proximate Southern Levant (Additional files 10 and 11).

### Genetic contribution from proximate wild populations into landraces

We determined the genetic contribution of wild populations in landraces for those growing in the same geographic range as the natural range of wild barley (Additional files 13 and 14). East African landraces are outside this range; therefore they were not considered in this analysis. We calculated the great circle distance between each landrace and the nearest wild individual from each wild population using the R package pracma [61]. We then calculated the correlation between distance and the proportion of ancestry assigned in SupportMix.

### Private/Shared alleles analysis

Using the 1,896 SNPs in common between landraces and wild barley, we identified alleles private to each of the five wild barley populations using the software SharedPoly from the libsequence library [56]. We found 115, 20, 20, 17, and nine private alleles corresponding to Southern Levant, Northern Levant, Central Asian, Northern Mesopotamia, and Syrian Desert wild barley populations, respectively (Additional file 16). We search for the presence of these SNPs that are private to individual wild populations in each of the landrace populations; this class of variants is referred to as shared alleles and their observed frequency in each landrace population is shown in Additional file 17 (see also Additional file 15). The estimation of frequency is based on diploid sample size, thus at a given SNP, heterozygous individuals contribute one allele private to the wild population analyzed, and homozygous sites are counted either as zero or two.

### Identity by State

An Identity by State (IBS) analysis between the wild and landrace barley lines is used to test for shared genomic segments between populations, consistent with recent introgression. The IBS analysis used PLINK v.1.90 [62] with window sizes of 30 SNPs. Larger window sizes resulted in no shared segments between these two datasets. Therefore, we report the results for windows of 30 SNPs. Only segments with 100 % match for the 30 SNPs were considered as significant. There are 37 non-overlapping IBS segments between landraces and wild, with 18 % of wild individuals sharing segments with 36 % of the landraces within each landrace population (Additional file 17). On average the IBS segments composed of 30 SNPs represent 10.48 cM genomic regions (Additional files 18 and 19).

All code used for analysis and for figures can be found in the GitHub repository, https://github.com/AnaPoets/BarleyLandraces. The raw genotyping data for the 2,446 accessions in the NSGC are available in Figshare, http://figshare.com/articles/Raw_Genotyping_Data_Barley_landraces_are_characterized_by_geographically_heterogeneous_genomic_origins/1468432.

## Additional files

Additional file 1: Table S1. 803 landrace accessions used in this study with latitude and longitude information. Additional file 2: Figure S1. Population structure of barley landraces. All clusters from *K* = 2 to 7: Central European, Southern European, Northern European, East African, the Himalayan Mountains, Himalayan Mountains and Middle Eastern, and Central Asian. The Y-axis is percent composition and the X-axis displays accessions sorted geographically from west to east. Additional file 3: Figure S2. Relationship of barley landrace accessions based on principal components. (A) Principal Component Analysis transformation of the genetic variation in barley landraces. Compares projected locations to sample localities as depicted in Fig 1, by rotating PC1 versus PC2 93° clockwise. (B) Principal Component Analysis transformation of the genetic variation in barley landraces. Compares projected locations to sample localities as depicted in Fig 1, by rotating PC1 versus PC3 70° clockwise. This comparison results in a greater separation of the East African population from other landrace populations. Additional file 4: Table S2. Median and maximum focal *F*_ST_ values from comparisons of each landrace population to all other landraces. Additional file 5: Figure S3. Population structure in wild barley. Each of the six colors represents one of the six subpopulations. Three different subpopulations are nested in the Eastern and Western populations, respectively. This figure has been reproduced from [14]. Additional file 6: Figure S4. Excess or deficit of ancestry for barley landrace populations. Excess or deficit (Δ ancestry) measured as the deviation from average contribution of each wild population from average genome-wide contributions (black dashed line). Colors correspond to the five populations identified in wild barley (Additional file 5). Positive values indicate an excess and negative values a deficit of ancestry from a particular wild population. The dotted horizontal line indicates the 98th percentile cutoff from the distribution of excess or deficit of each wild population across all genomic segments for each landrace population. Additional file 7: Table S3. Chromosome painting. Individual landrace ancestry inferred at each genomic region. The cells are colored according to their inferred ancestry from the wild populations. Two haplotypes (rows) per landrace accession are depicted. Additional file 8: Figure S5. Proportion of ancestry in barley landrace populations at each genomic segment. Ancestry proportions include unassigned sites. (A) Central European landrace population, (B) Asian landrace population, (C) Coastal Mediterranean landrace population, (D) East African landrace population. The tick marks on the x-axis in panel D indicate the linkage group boundaries. Additional file 9: Table S4 Genome-wide proportion of ancestry among each landrace population. Genome-wide average proportions of genetic ancestry in barley landrace populations for all regions that have a probability of assignment >95 %. Additional file 10: Figure S6 Predictive accuracy of SupportMix by cross-validation. Each panel represents the average proportion of ancestry assigned to individuals from a wild population used as a test dataset compared to a training dataset composed of all remaining wild barley individuals. The analysis was run 50 times for four individuals from each wild population (proportions represent only sites with assigned ancestry). Additional file 11: Table S5. Predictive accuracy of SupportMix by cross-validation. Averaged across 50 runs of the genome-wide proportion of ancestry in subsets of wild barley analyzed as testing samples, using the remaining wild individuals as the validation dataset. Additional file 12: Figure S7. Distribution of the genome-wide proportion of ancestry from wild to landrace barley populations (unassigned genomic regions are not considered). Additional file 13: Table S6. Genome-wide ancestry as a function of distance from wild populations. Proportions of ancestry for individual landraces, and the great circle distance between each individual and the closest accession from each wild population. Additional file 14: Figure S8. Genome-wide ancestry as a function of distance from wild populations. The map on the bottom right shows the distribution of landraces sampled from within the natural range of wild barley. The boxes represent the geographic distribution of Southern Levant (blue), Northern Mesopotamia (red), Syrian Desert (green), Northern Levant (orange), and Central Asian (purple) wild populations. The other panels indicate the distribution of proportion of ancestry (Y-axis) in each of the landrace populations as a function of distance (X-axis) from the ancestral wild population. The boxplots for each landrace population are at the median of the distribution of distances calculated for each landrace and the closest wild accession (depicted at coordinates 0,0). The correlation (*r*) between distance and proportion of ancestry is indicated in each comparison. East African landraces are not included in the depiction due to small sample size (two individuals) in the geographic range analyzed. Additional file 15: Table S7. Frequency of alleles private to the wild present in each of the landrace populations. Private SNPs in wild barley that are present in the landraces, including linkage group and their frequency in each landrace population. Additional file 16: Table S8. Summary of number of private alleles from wild barley populations present in the landraces. Average allele frequency of alleles private to wild populations in the landraces (in parenthesis). The number of private alleles in each wild population is shown in brackets. Additional file 17: Figure S9. Frequency of alleles private to the wild populations present in each of the landrace populations. Linkage groups are separated by gray dashes. Additional file 18: Figure S10. Identical by State segments between wild and cultivated barley. Black dots represent SNPs in each landrace population. The x-axis is the genomic location of each SNP. The vertical gray dashed lines define the limits between linkage groups. The colored lines represent the location and extend of IBS between each wild and landrace population. Each segment is 30 SNPs long. Additional file 19: Table S9. Proportion of individuals involved in IBS segments (30 SNP each) between each landrace population and the 277 wild barley lines. Additional file 20: Figure S11. Linkage disequilibrium (*r* ^2^). Linkage disequilibrium determined by a pairwise comparison of the SNPs in each linkage group in the landraces. Additional file 21: Table S10. 1,896 SNPs shared between wild barley and landrace populations. Additional file 22: Figure S12. Identification of the optimal number of groups *K*. (A) ΔK, description of the four steps to determine the number of clusters that best explain the population structure among the landraces; (B) Clusterdness, the extent to which individuals were estimated to belong to a single cluster rather than to a combination of clusters. Additional file 23: Figure S13. Maximum Likelihood tree among wild (bold font) and barley landraces as inferred by TreeMix.
