# Supplementary figures and images for: Barley landraces are characterized by geographically heterogeneous genomic origins
Source: Genome Biol. 2015 Aug 21;16(1):173. doi: 10.1186/s13059-015-0712-3 (PMC4546095; doi:10.1186/s13059-015-0712-3)

Proportion of assignment

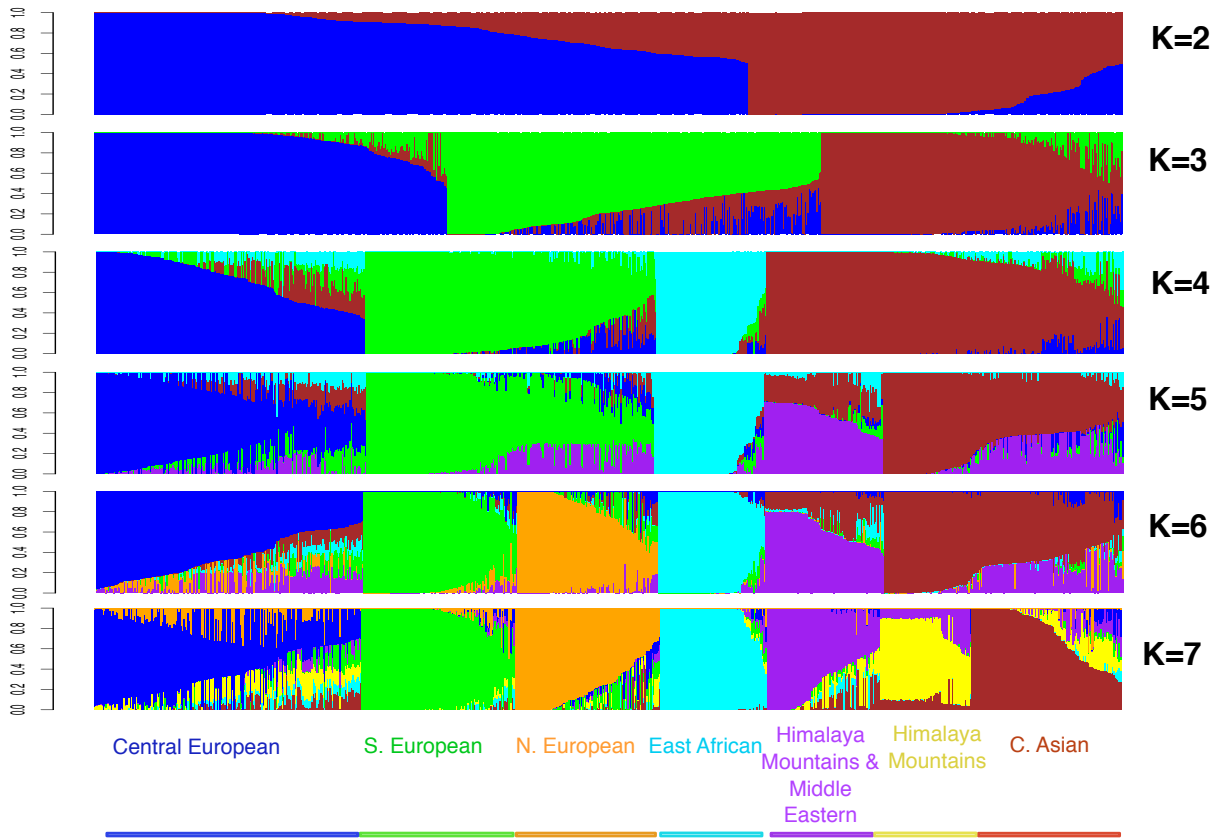

Supplement: Additional file 2: Figure S1. — Population structure of barley landraces. All clusters from K = 2 to 7: Central European, Southern European, Northern European, East African, the Himalayan Mountains, Himalayan Mountains and Middle Eastern, and Central Asian. The Y-axis is percent composition and the X-axis displays accessions sorted geographically from west to east. [file 13059_2015_712_MOESM2_ESM.pdf]

**A**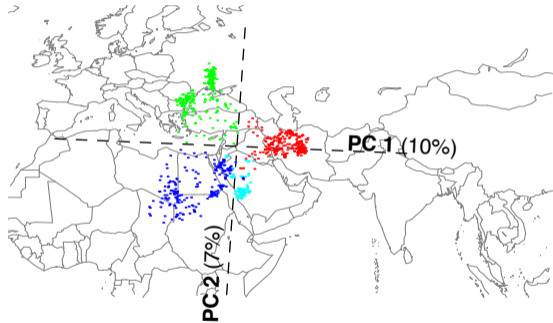**B**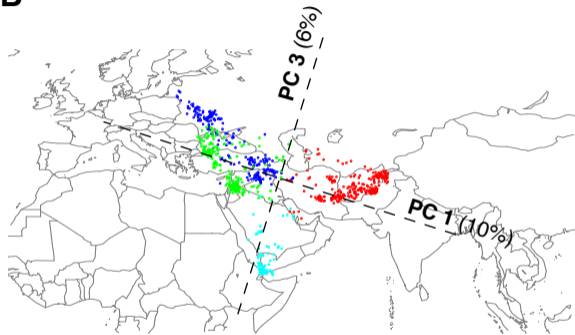

Supplement: Additional file 3: Figure S2. — Relationship of barley landrace accessions based on principal components. (A) Principal Component Analysis transformation of the genetic variation in barley landraces. Compares projected locations to sample localities as depicted in Fig 1, by rotating PC1 versus PC2 93° clockwise. (B) Principal Component Analysis transformation of the genetic variation in barley landraces. Compares projected locations to sample localities as depicted in Fig 1, by rotating PC1 versus PC3 70° clockwise. This comparison results in a greater separation of the East African population from other landrace populations. [file 13059_2015_712_MOESM3_ESM.pdf]

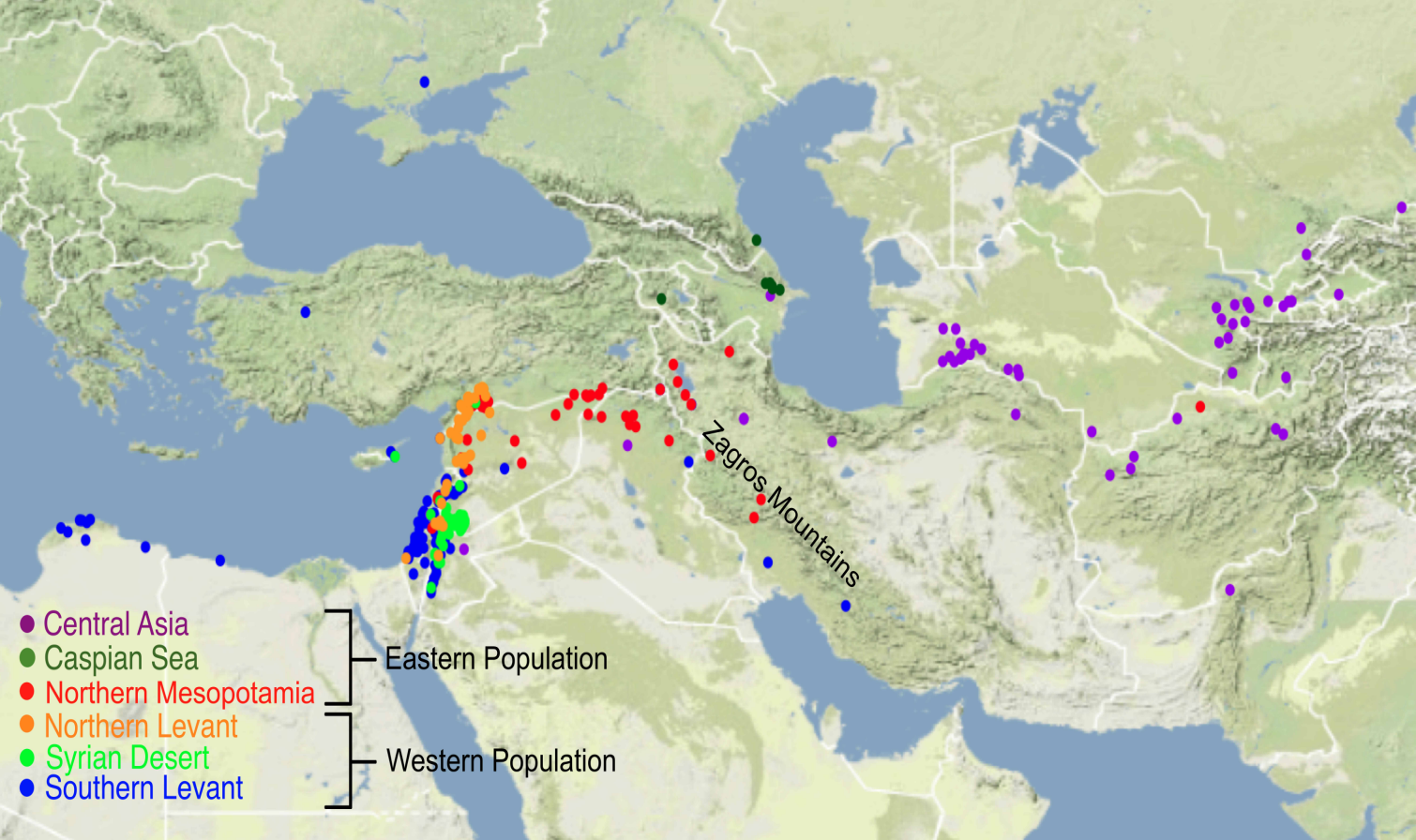

Supplement: Additional file 5: Figure S3. — Population structure in wild barley. Each of the six colors represents one of the six subpopulations. Three different subpopulations are nested in the Eastern and Western populations, respectively. This figure has been reproduced from [14]. [file 13059_2015_712_MOESM5_ESM.pdf]

**A**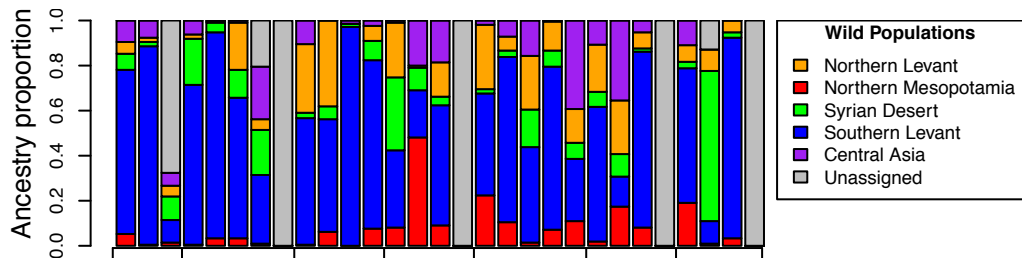**B**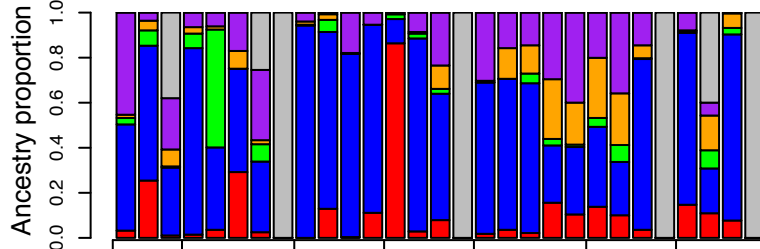**C**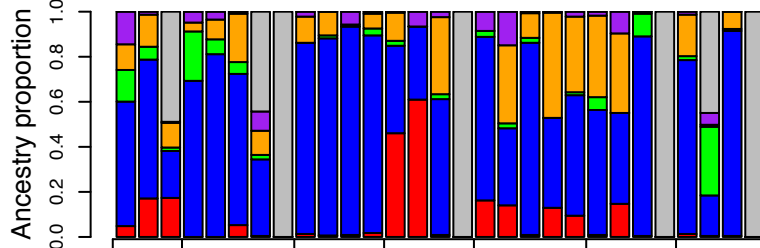**D**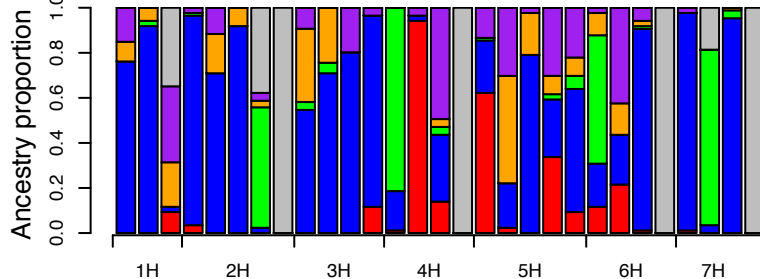

Supplement: Additional file 8: Figure S5. — Proportion of ancestry in barley landrace populations at each genomic segment. Ancestry proportions include unassigned sites. (A) Central European landrace population, (B) Asian landrace population, (C) Coastal Mediterranean landrace population, (D) East African landrace population. The tick marks on the x-axis in panel D indicate the linkage group boundaries. [file 13059_2015_712_MOESM8_ESM.pdf]

# IBS segments between wild and barley landraces

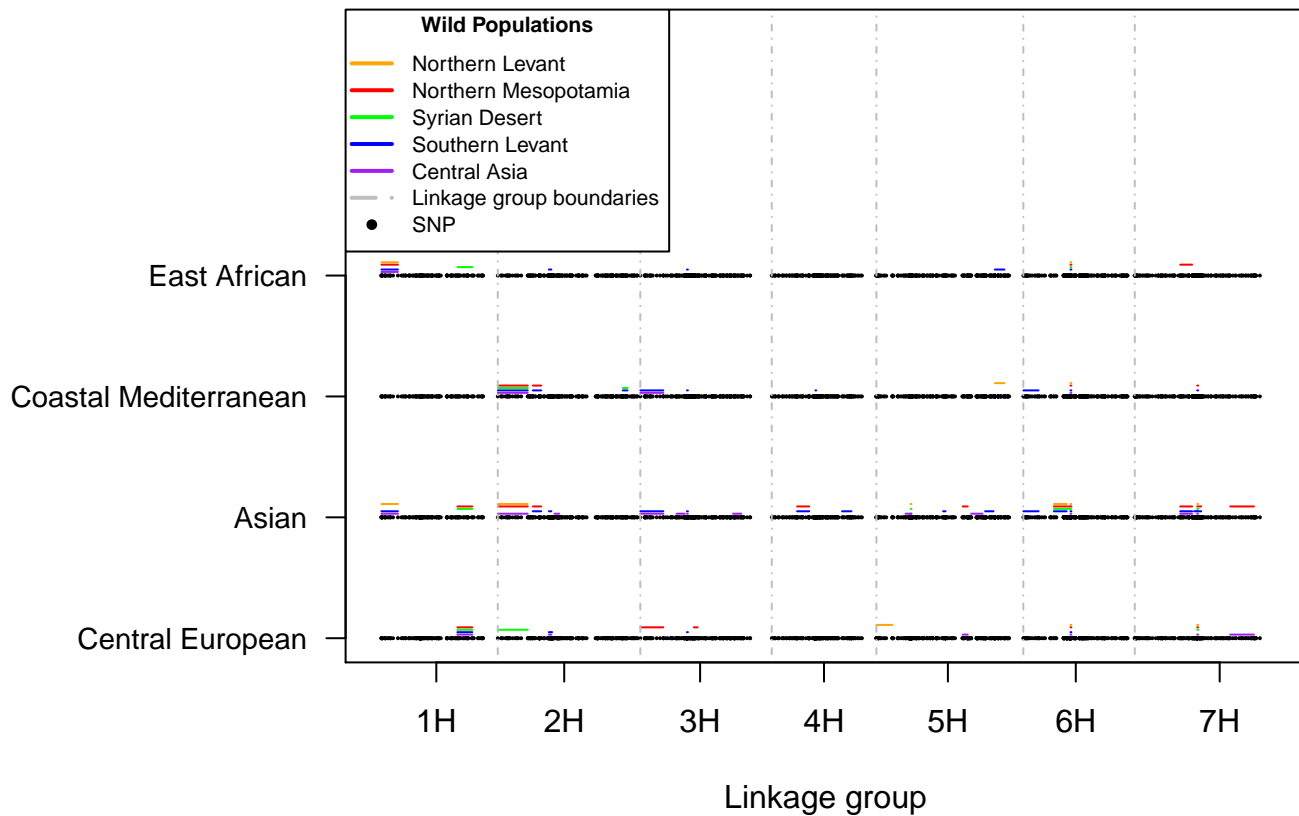

Supplement: Additional file 18: Figure S10. — Identical by State segments between wild and cultivated barley. Black dots represent SNPs in each landrace population. The x-axis is the genomic location of each SNP. The vertical gray dashed lines define the limits between linkage groups. The colored lines represent the location and extend of IBS between each wild and landrace population. Each segment is 30 SNPs long. [file 13059_2015_712_MOESM18_ESM.pdf]

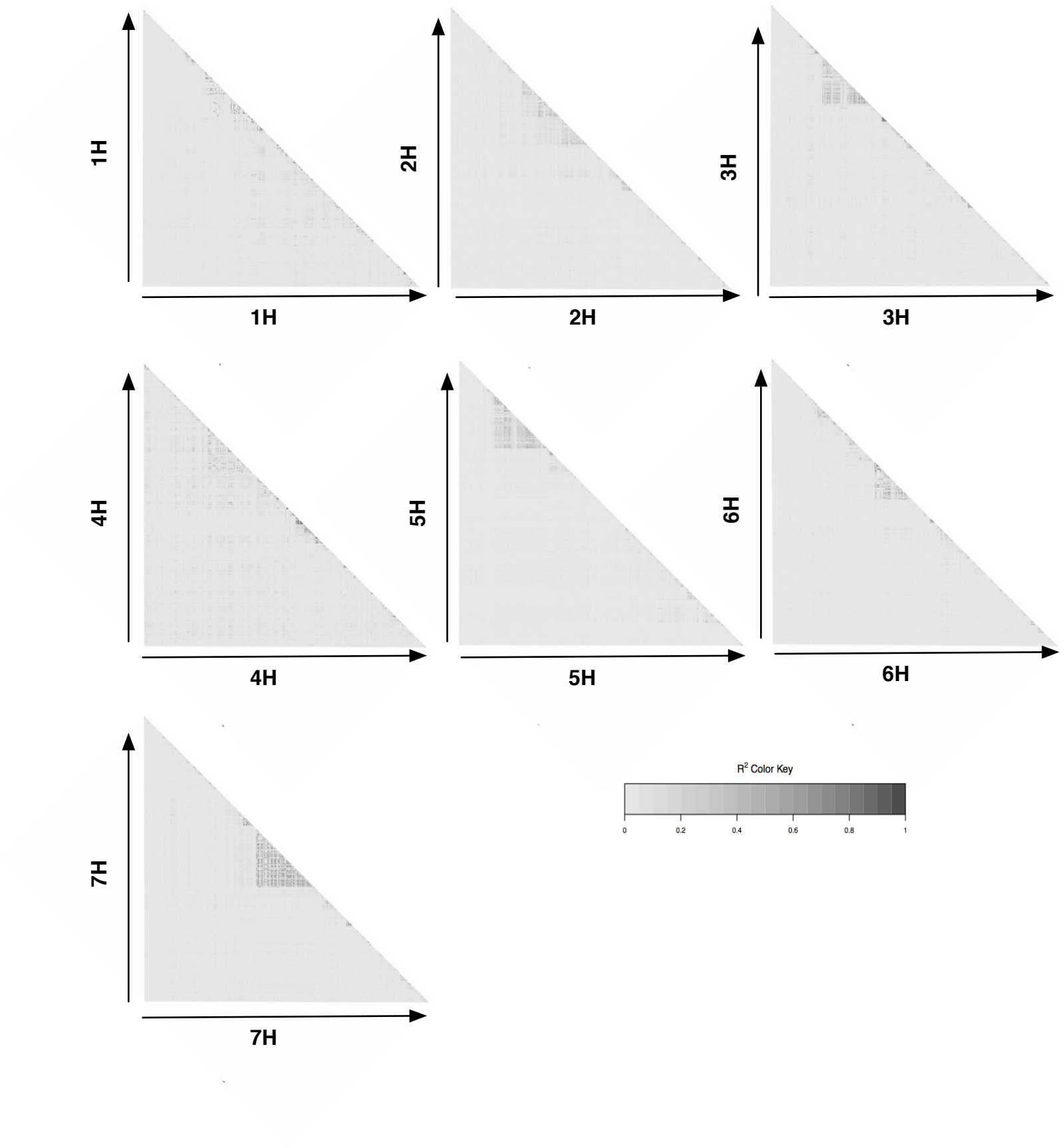

Supplement: Additional file 20: Figure S11. — Linkage disequilibrium (r 2). Linkage disequilibrium determined by a pairwise comparison of the SNPs in each linkage group in the landraces. [file 13059_2015_712_MOESM20_ESM.pdf]

**A**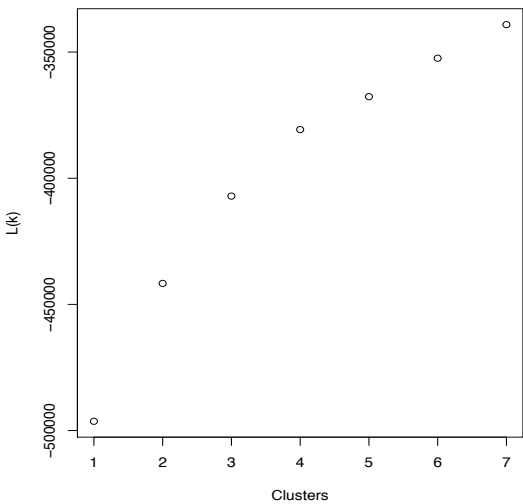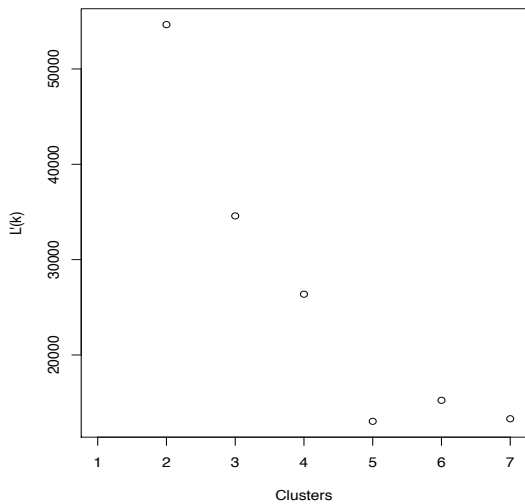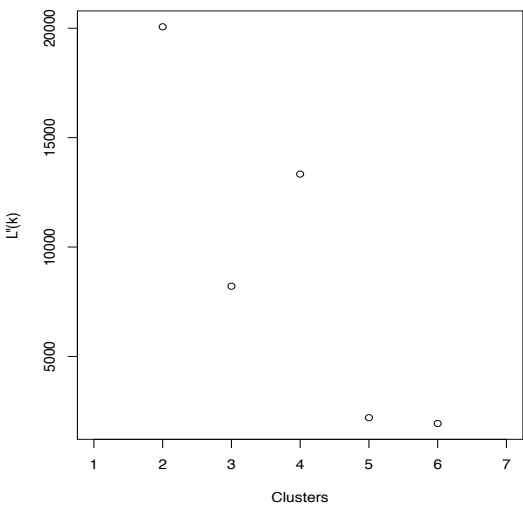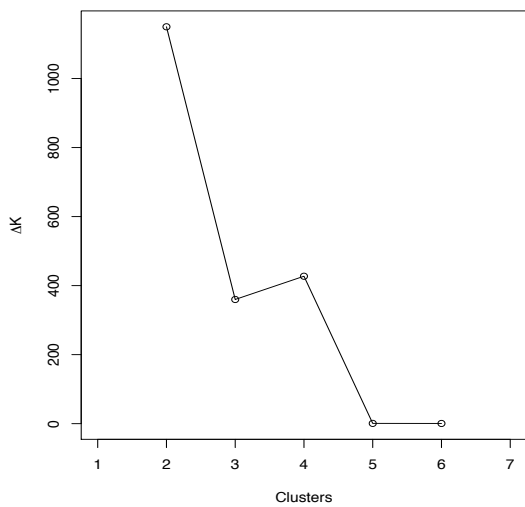**B**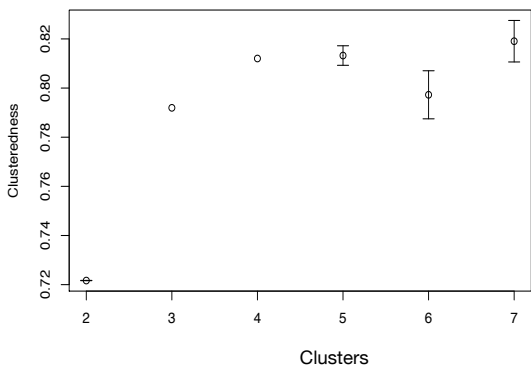

Supplement: Additional file 22: Figure S12. — Identification of the optimal number of groups K. (A) ΔK, description of the four steps to determine the number of clusters that best explain the population structure among the landraces; (B) Clusterdness, the extent to which individuals were estimated to belong to a single cluster rather than to a combination of clusters. [file 13059_2015_712_MOESM22_ESM.pdf]

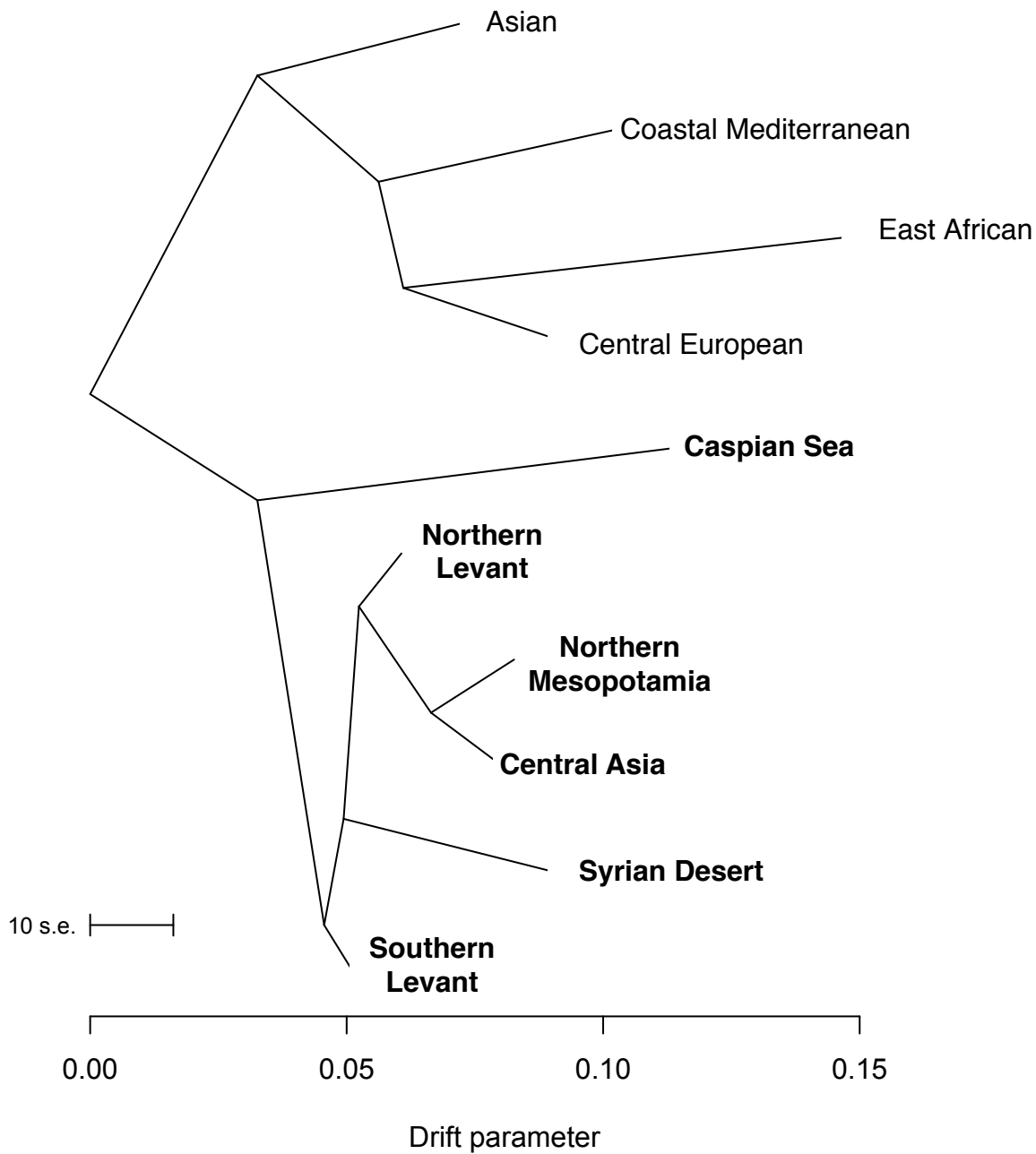

Supplement: Additional file 23: Figure S13. — Maximum Likelihood tree among wild (bold font) and barley landraces as inferred by TreeMix. [file 13059_2015_712_MOESM23_ESM.pdf]
