# Supplementary material for: Barley landraces are characterized by geographically heterogeneous genomic origins
Source: Genome Biol. 2015 Aug 21;16(1):173. doi: 10.1186/s13059-015-0712-3 (PMC4546095; doi:10.1186/s13059-015-0712-3)
Supplement: Additional file 6: Figure S4. — Excess or deficit of ancestry for barley landrace populations. Excess or deficit (Δ ancestry) measured as the deviation from average contribution of each wild population from average genome-wide contributions (black dashed line). Colors correspond to the five populations identified in wild barley (Additional file 5). Positive values indicate an excess and negative values a deficit of ancestry from a particular wild population. The dotted horizontal line indicates the 98th percentile cutoff from the distribution of excess or deficit of each wild population across all genomic segments for each landrace population. [file 13059_2015_712_MOESM6_ESM.pdf]

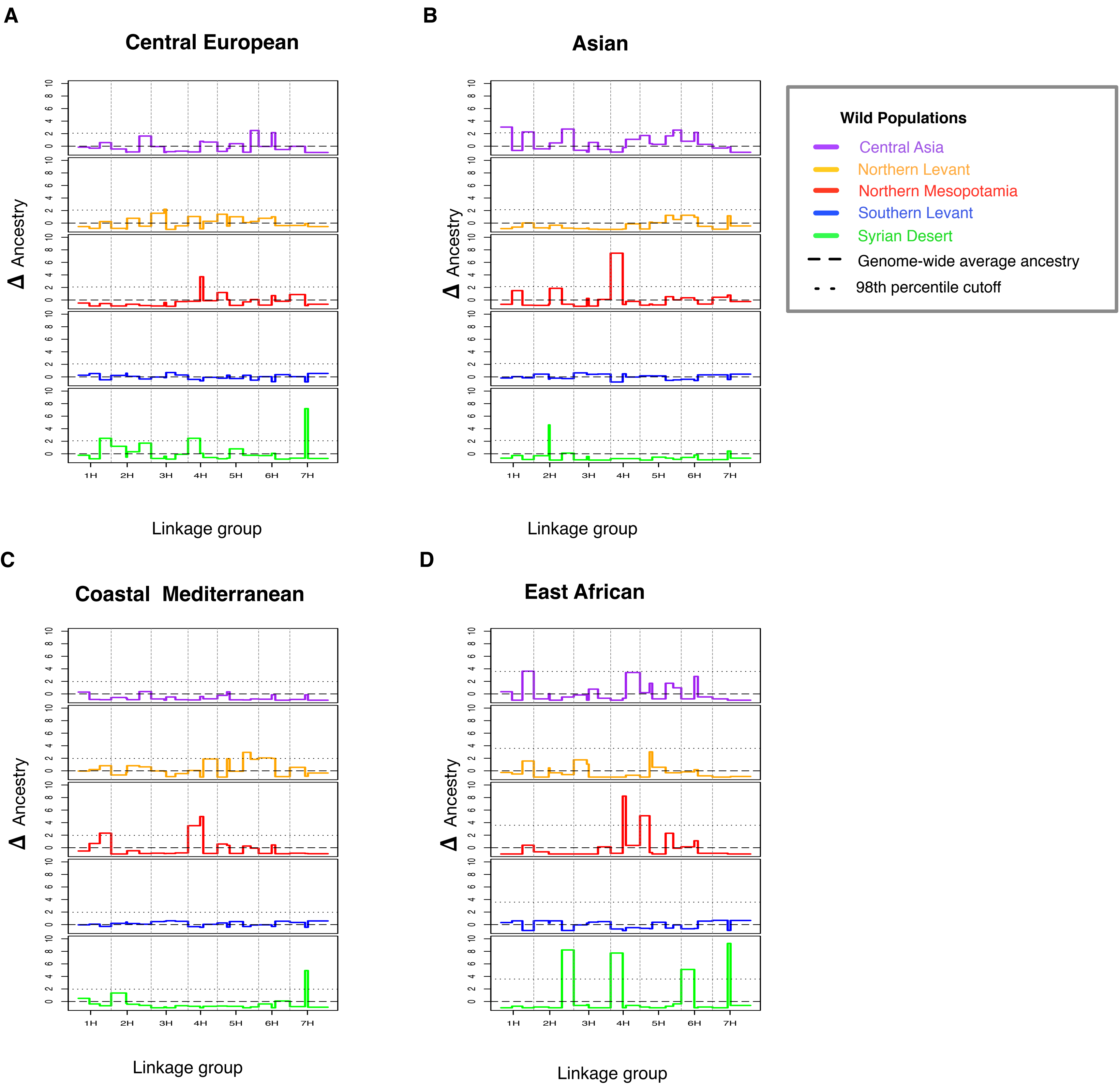

A
Central European
B
Asian
C
Coastal Mediterranean
D
East African
Δ Ancestry
Linkage group
1H
2H
3H
4H
5H
6H
7H
Wild Populations
Central Asia
Northern Levant
Northern Mesopotamia
Southern Levant
Syrian Desert
Genome-wide average ancestry
98th percentile cutoff
