# Supplementary material for: Barley landraces are characterized by geographically heterogeneous genomic origins
Source: Genome Biol. 2015 Aug 21;16(1):173. doi: 10.1186/s13059-015-0712-3 (PMC4546095; doi:10.1186/s13059-015-0712-3)
Supplement: Additional file 10: Figure S6 — Predictive accuracy of SupportMix by cross-validation. Each panel represents the average proportion of ancestry assigned to individuals from a wild population used as a test dataset compared to a training dataset composed of all remaining wild barley individuals. The analysis was run 50 times for four individuals from each wild population (proportions represent only sites with assigned ancestry). [file 13059_2015_712_MOESM10_ESM.pdf]

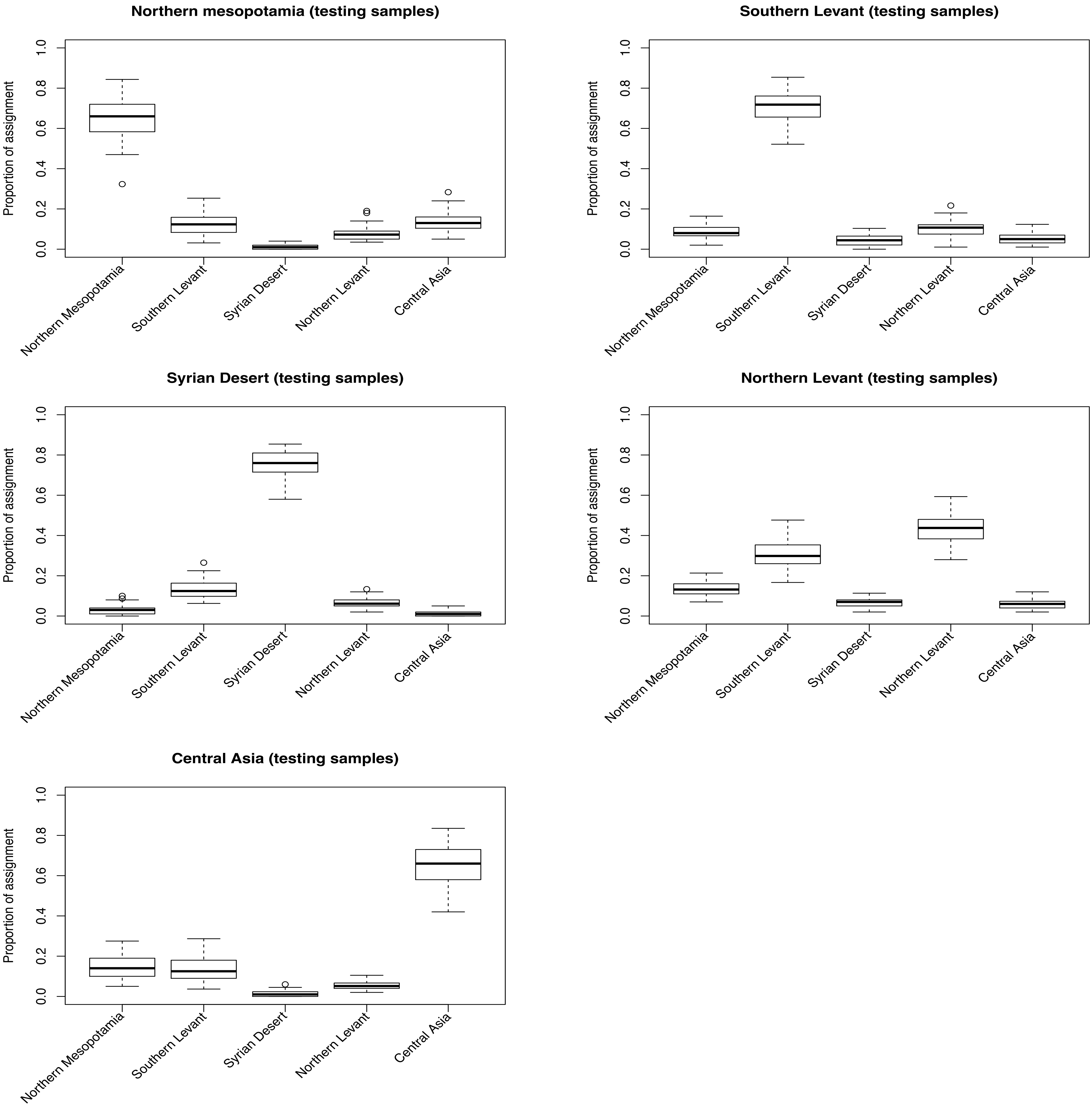
Northern mesopotamia (testing samples)
Southern Levant (testing samples)
Syrian Desert (testing samples)
Northern Levant (testing samples)
Central Asia (testing samples)
Proportion of assignment
0.0
0.2
0.4
0.6
0.8
1.0
Northern Mesopotamia
Southern Levant
Syrian Desert
Northern Levant
Central Asia
