# Supplementary material for: Barley landraces are characterized by geographically heterogeneous genomic origins
Source: Genome Biol. 2015 Aug 21;16(1):173. doi: 10.1186/s13059-015-0712-3 (PMC4546095; doi:10.1186/s13059-015-0712-3)
Supplement: Additional file 12: Figure S7. — Distribution of the genome-wide proportion of ancestry from wild to landrace barley populations (unassigned genomic regions are not considered). [file 13059_2015_712_MOESM12_ESM.pdf]

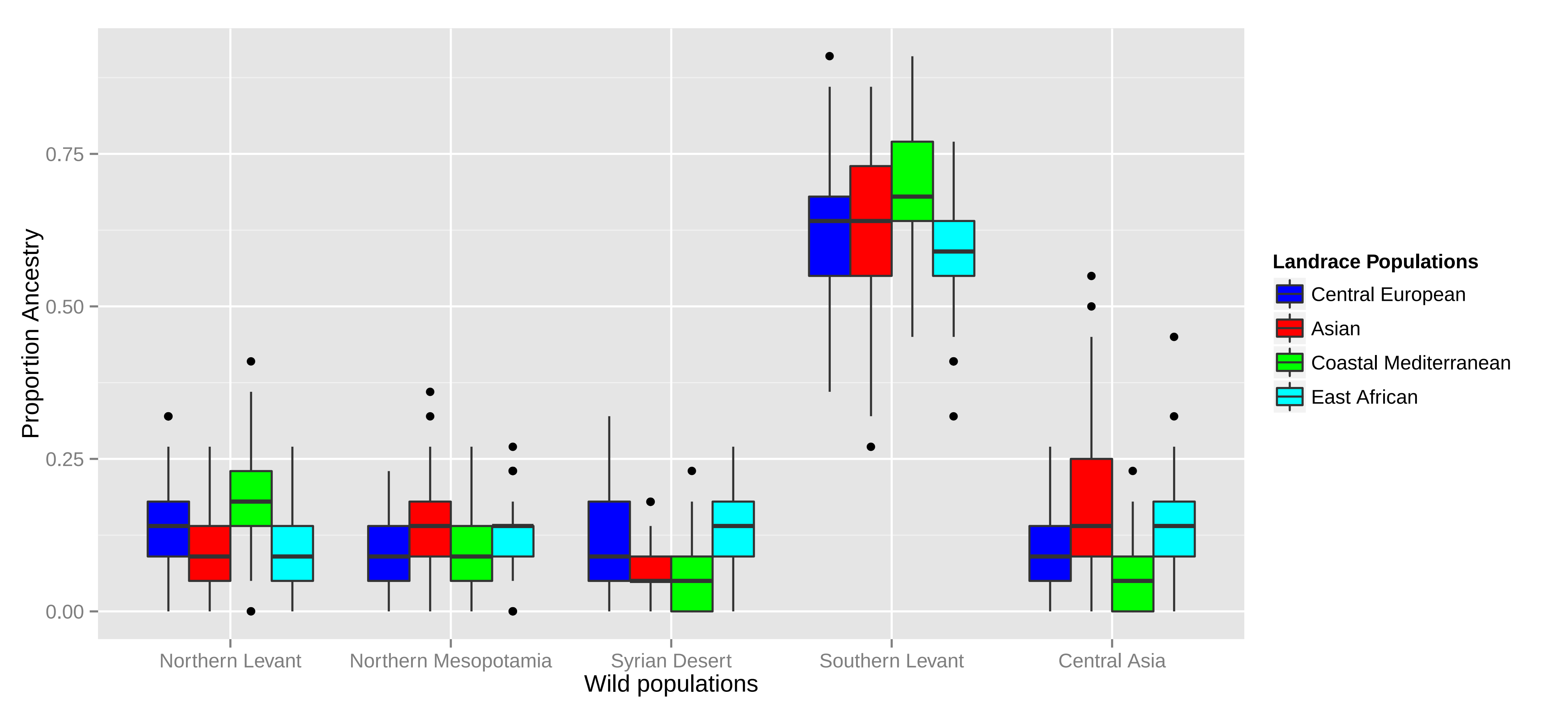

0.75
0.50
0.25
0.00
Proportion Ancestry
Northern Levant
Northern Mesopotamia
Syrian Desert
Southern Levant
Central Asia
Wild populations
Landrace Populations
Central European
Asian
Coastal Mediterranean
East African
