# Supplementary material for: Barley landraces are characterized by geographically heterogeneous genomic origins
Source: Genome Biol. 2015 Aug 21;16(1):173. doi: 10.1186/s13059-015-0712-3 (PMC4546095; doi:10.1186/s13059-015-0712-3)
Supplement: Additional file 14: Figure S8. — Genome-wide ancestry as a function of distance from wild populations. The map on the bottom right shows the distribution of landraces sampled from within the natural range of wild barley. The boxes represent the geographic distribution of Southern Levant (blue), Northern Mesopotamia (red), Syrian Desert (green), Northern Levant (orange), and Central Asian (purple) wild populations. The other panels indicate the distribution of proportion of ancestry (Y-axis) in each of the landrace populations as a function of distance (X-axis) from the ancestral wild population. The boxplots for each landrace population are at the median of the distribution of distances calculated for each landrace and the closest wild accession (depicted at coordinates 0,0). The correlation (r) between distance and proportion of ancestry is indicated in each comparison. East African landraces are not included in the depiction due to small sample size (two individuals) in the geographic range analyzed. [file 13059_2015_712_MOESM14_ESM.pdf]

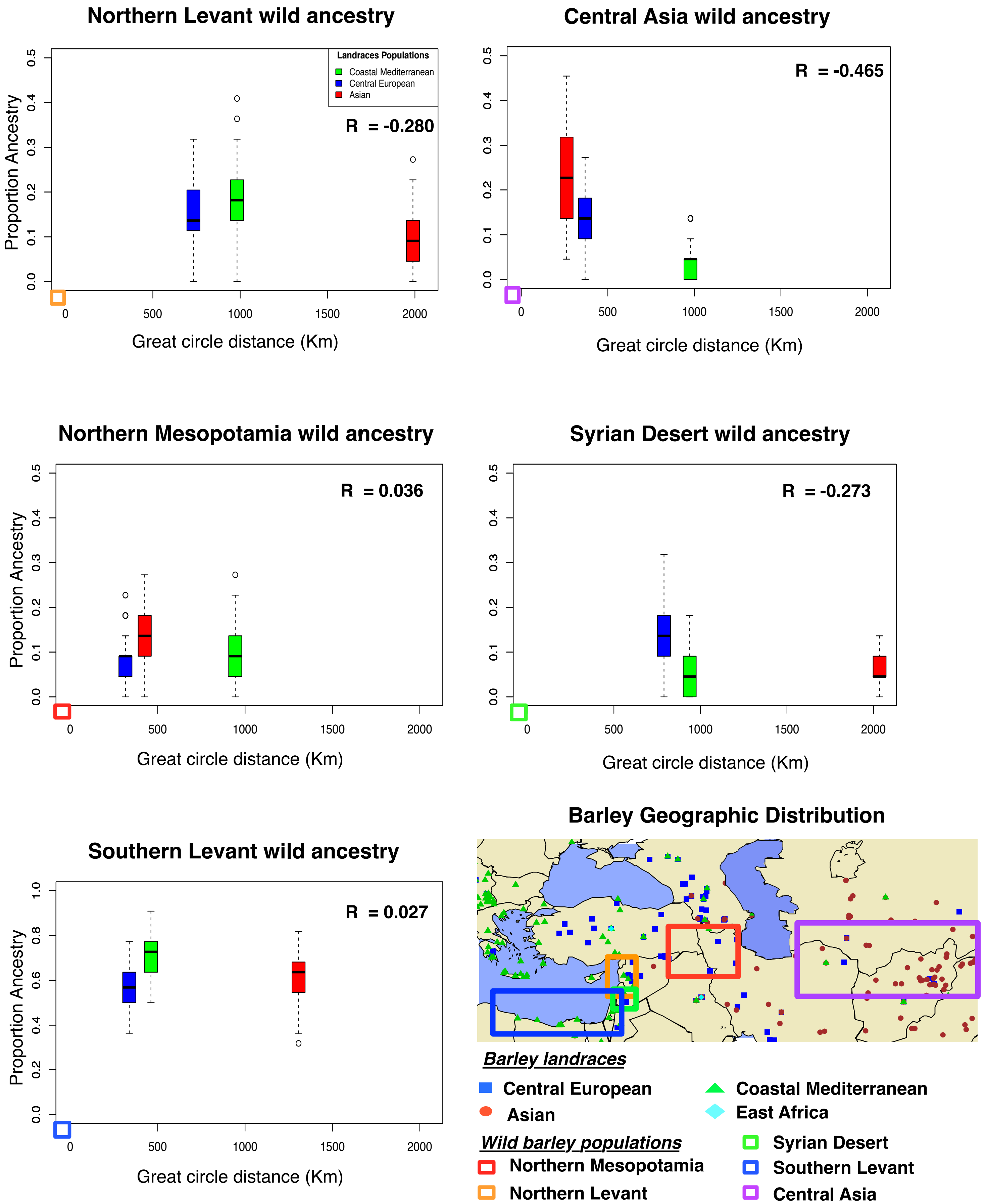
Northern Levant wild ancestry
Landraces Populations
Coastal Mediterranean
Central European
Asian
R = -0.280
Proportion Ancestry
0.0
0.1
0.2
0.3
0.4
0.5
0
500
1000
1500
2000
Great circle distance (Km)
Central Asia wild ancestry
R = -0.465
0.0
0.1
0.2
0.3
0.4
0.5
0
500
1000
1500
2000
Great circle distance (Km)
Northern Mesopotamia wild ancestry
R = 0.036
Proportion Ancestry
0.0
0.1
0.2
0.3
0.4
0.5
0
500
1000
1500
2000
Great circle distance (Km)
Syrian Desert wild ancestry
R = -0.273
0.0
0.1
0.2
0.3
0.4
0.5
0
500
1000
1500
2000
Great circle distance (Km)
Southern Levant wild ancestry
R = 0.027
Proportion Ancestry
0.0
0.2
0.4
0.6
0.8
1.0
0
500
1000
1500
2000
Great circle distance (Km)
Barley Geographic Distribution
Barley landraces
Central European
Coastal Mediterranean
Asian
East Africa
Wild barley populations
Syrian Desert
Northern Mesopotamia
Southern Levant
Northern Levant
Central Asia
