# Supplementary material for: Barley landraces are characterized by geographically heterogeneous genomic origins
Source: Genome Biol. 2015 Aug 21;16(1):173. doi: 10.1186/s13059-015-0712-3 (PMC4546095; doi:10.1186/s13059-015-0712-3)
Supplement: Additional file 17: Figure S9. — Frequency of alleles private to the wild populations present in each of the landrace populations. Linkage groups are separated by gray dashes. [file 13059_2015_712_MOESM17_ESM.pdf]

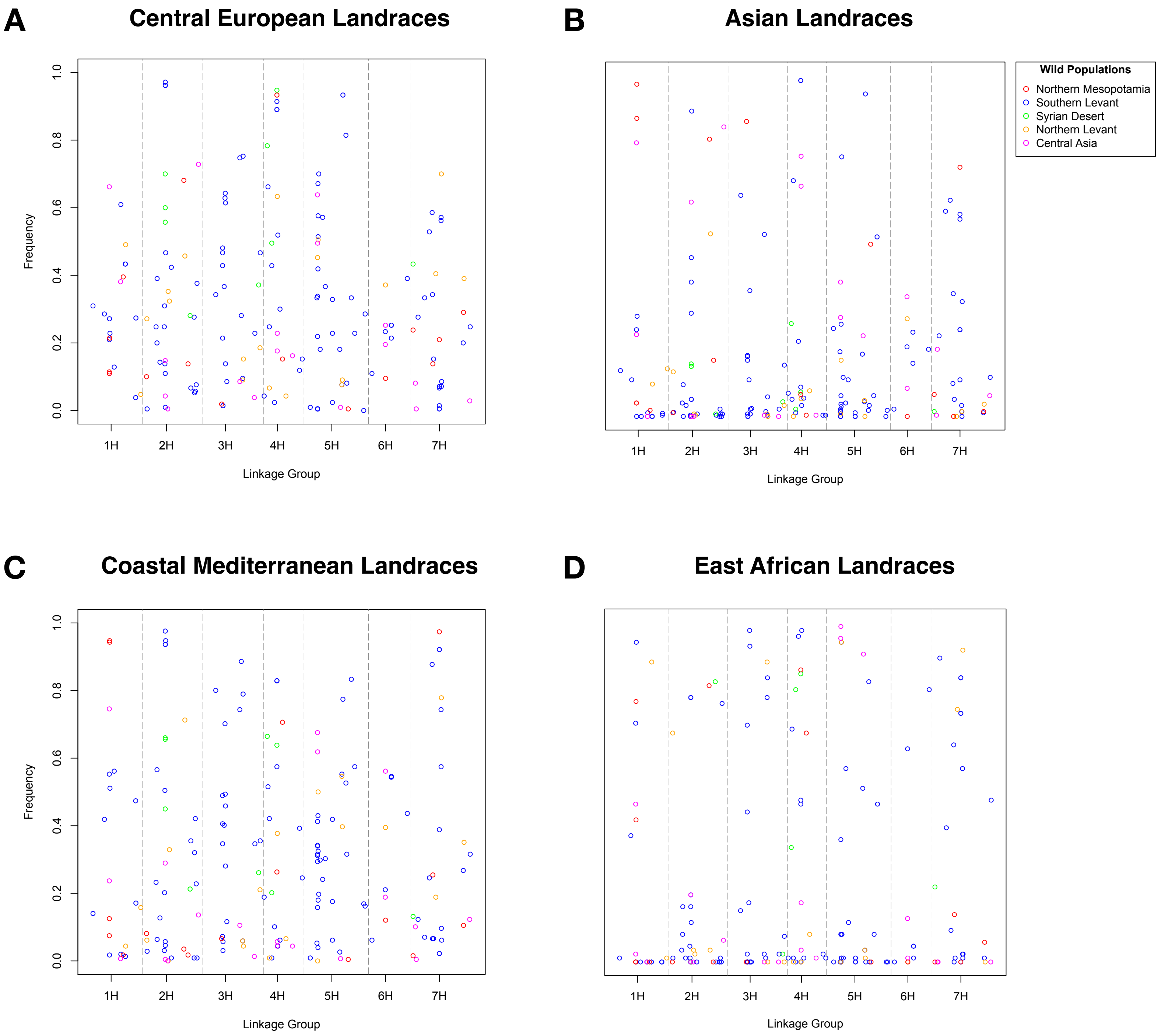
A
Central European Landraces
B
Asian Landraces
C
Coastal Mediterranean Landraces
D
East African Landraces
Frequency
0.0
0.2
0.4
0.6
0.8
1.0
1H
2H
3H
4H
5H
6H
7H
Linkage Group
Wild Populations
Northern Mesopotamia
Southern Levant
Syrian Desert
Northern Levant
Central Asia
